# Enteroviral Meningoencephalitis as a Complication of Rituximab Therapy for Rheumatoid Arthritis

**DOI:** 10.7759/cureus.18189

**Published:** 2021-09-22

**Authors:** Samuel G Cook, Aven W Ford, David A Lindholm, Joshua Scott

**Affiliations:** 1 Department of Internal Medicine, Wright State University Boonshoft School of Medicine, Dayton, USA; 2 Department of Medicine, Wright Patterson Medical Center, Wright Patterson AFB, USA

**Keywords:** ivig, rheumatoid arthritis, rituximab, meningoencephalitis, enterovirus

## Abstract

Enteroviral meningoencephalitis is a rare complication of rituximab therapy that has been described in the treatment of hematologic malignancies. We report the first case of enteroviral meningoencephalitis in a patient receiving rituximab for the treatment of rheumatoid arthritis.

A 37-year-old female treated with rituximab for severe rheumatoid arthritis presented with fever, headache, confusion, and tremor. Magnetic resonance imaging (MRI) of the brain was unrevealing. Cerebrospinal fluid showed a lymphocytic pleocytosis and multiplex polymerase chain reaction (PCR) was positive for enterovirus. She was treated with intravenous immunoglobulin (IVIG) for five days and had significant improvement in symptoms.

Rituximab is an anti-CD20 monoclonal antibody that induces B-cell depletion and possible hypogammaglobulinemia, putting patients at increased risk for viral infections. We present this case to highlight that patients on rituximab, regardless of the indication for use, are susceptible to severe complications of otherwise typically self-limited viral infections. Furthermore, we describe the potential use of IVIG in treating these patients.

## Introduction

Enteroviruses are the most common cause of viral meningitis, with most cases occurring in children and resulting in complete recovery [1,2]. However, enteroviral meningoencephalitis can be fatal. This is typically observed in patients with congenital humoral immunodeficiencies, although cases have been described as a rare infectious complication of rituximab [3]. Rituximab was initially used in the treatment of lymphoma but was later approved for use in rheumatoid arthritis that had an inadequate response to tumor necrosis factor (TNF) antagonists [4]. Here, we report the first case of enteroviral meningoencephalitis in a patient receiving rituximab for the treatment of rheumatoid arthritis.

## Case presentation

**Figure 1:**
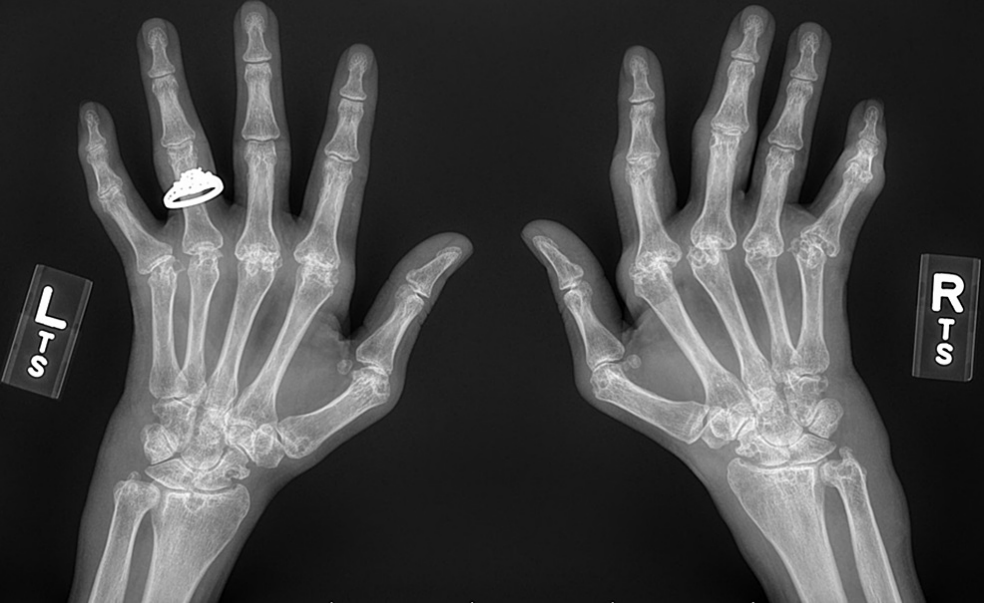
Bilateral hand x-ray demonstrating erosive arthritis, most notable in the metacarpophalangeal joints and wrists, along with ulnar deviation of the digits.

A 37-year-old female with a history of seropositive, erosive, treatment-resistant rheumatoid arthritis, was undergoing treatment with rituximab. Her initial diagnosis of rheumatoid arthritis was 15 years prior to this presentation. She maintained a high disease activity despite trials of multiple therapies, eventually developing erosions and deformities of the hands (Figure 1). Her prior medications included adalimumab, etanercept, tocilizumab, infliximab, abatacept, and tofacitinib, none of which achieved low disease activity. She was eventually maintained on combination therapy with methotrexate, leflunomide, hydroxychloroquine, and rituximab. She had been on rituximab for three years prior to this presentation, administered as two 1,000 mg infusions 14 days apart every six months. Her most recent infusion was six weeks prior to the presentation described below.

The patient presented to her primary care clinic with one week of fever, sore throat, and a painful tongue ulcer. There were no known sick contacts. She received cephalexin for empiric treatment of presumed streptococcal pharyngitis. Five days later, she developed ear pain and tinnitus, for which she was again treated with cephalexin for presumed otitis media. Over the next three days, she reported worsening fevers, headache, dizziness, confusion, and tremors, prompting presentation to the emergency department. In the emergency department, a physical exam was notable for disorientation to time, facial and upper extremity tremor, and antalgic gait. Initial laboratory workup included complete blood count, metabolic panel, erythrocyte sedimentation rate, and c-reactive protein, which were all within normal limits. Magnetic resonance imaging (MRI) of the brain with and without contrast showed no acute intracranial pathology or abnormal enhancement. Cerebrospinal fluid (CSF) analysis revealed normal protein, normal glucose, and a lymphocytic pleocytosis, with 31 cells/mcL and 83% lymphocytes (Table 1). Commercial multiplex CSF polymerase chain reaction (PCR) detected enterovirus. Immunoglobulin panel was notable for hypogammaglobulinemia, with an immunoglobulin G (IgG) level of 489 mg/dL (reference range 600-1,600 mg/dL).

**Table 1:** Results of cerebrospinal fluid analysis (all tubes drawn from one lumbar puncture). dL = deciliter, mcL = microliter, mg = milligram, PCR = polymerase chain reaction, RBC = red blood cell, WBC = white blood cell

CSF Tube	Laboratory Parameter	Result	Reference
Tube 1	WBC	31 cells/mcL	0-5 cells/mcL
	RBC	22 cells/mcL	0-5 cells/mcL
	Neutrophils	0%	
	Lymphocytes	83%	
	Monocytes/Macrophages	17%	
Tube 2	Protein	42.2 mg/dL	15-45 mg/dL
	Glucose	54.0 mg/dL	40-70 mg/dL
Tube 3	Gram Stain	No organisms	
	Culture	No growth	
	Multiplex PCR	Enterovirus	
Tube 4	WBC	27 cells/mcL	0-5 cells/mcL
	RBC	0 cells/mcL	0-5 cells/mcL
	Neutrophils	8%	
	Lymphocytes	69%	
	Monocytes/Macrophages	23%	

She had progressive neurologic deterioration, with the development of agitation, delirium, and aphasia. Rheumatology, infectious disease, and neurology were consulted to assist with management. She was treated with intravenous immunoglobulin (IVIG) 0.4g/kg daily for five days. This resulted in significant improvement of her agitation, confusion, tremor, gait, and headaches. On day 7 after admission, she was fully oriented but still struggled with concentration, memory, and emotional lability. Her symptoms completely resolved over the following 12 months.

## Discussion

Rituximab is a monoclonal antibody approved for the treatment of non-Hodgkin lymphomas, chronic lymphocytic leukemia, granulomatosis with polyangiitis, microscopic polyangiitis, pemphigus vulgaris, and rheumatoid arthritis [4]. It targets the CD20 antigen on B-lymphocytes, resulting in B-cell depletion and occasional hypogammaglobulinemia for 6-12 months after administration [5].

B cells are an essential component of humoral immunity that produces antibodies that neutralize virions and initiate the destruction of infected cells, effectively preventing the cellular spread of viruses [6]. The inhibition of the humoral immune response puts the patient at increased risk for viral infections, as evidenced by the black box warnings for reactivation of hepatitis B virus and infection with the John Cunningham (JC) virus [4]. There have been few reported cases of enteroviral meningoencephalitis associated with rituximab, the majority of which occurred in the treatment of hematologic disease [7-11]. A review of the literature indicates that our patient is the first reported case to occur in the treatment of rheumatoid arthritis, the only inflammatory arthritis for which rituximab has an approved indication.

The nonspecific clinical syndrome of headache, lethargy, tremor, and weakness can vary in severity and time from rituximab exposure [12]. CSF analysis usually demonstrates lymphocytic pleocytosis consistent with a viral etiology [1,2]. As with our patients, most cases are diagnosed with enterovirus PCR of the CSF. However, other cases have also been diagnosed via brain biopsy after non-diagnostic PCR [3].

While there is no standard treatment for enteroviral meningoencephalitis, IVIG has been used in previous cases. Treatment outcomes are variable ranging from resolution of neurologic symptoms to progression of disease and death [13,14]. There are several possible reasons for the wide spectrum, including delays in diagnosis due to nonspecific disease presentation as well as differences in the quantitative content of enteroviral antibodies between IVIG preparations [15]. Our patient had rapid improvement and eventual complete resolution of her symptoms following the use of IVIG.

## Conclusions

We present this case to highlight the risk that patients on rituximab, regardless of the indication for use, are susceptible to the severe complications of enterovirus, an otherwise typically self-limited pathogen. Clinicians should counsel their patients on this risk and maintain a high index of suspicion for these complications.
